# Guanxinning attenuates diabetic myocardial ischemia–reperfusion injury by targeting oral *Fusobacterium nucleatum* and modulating PTEN signaling

**DOI:** 10.3389/fphar.2025.1581413

**Published:** 2025-06-19

**Authors:** Yiwen Li, Qian Xu, Yanfei Liu, Longkun Liu, Wenting Wang, Mengmeng Zhu, Jing Cui, Hongjun Yang, Yue Liu

**Affiliations:** ^1^ Beijing Key Laboratory of Traditional Chinese Medicine Basic Research on Prevention and Treatment for Major Diseases Experimental Research Center China Academy of Chinese Medical Sciences, Beijing, China; ^2^ National Clinical Research Center for TCM Cardiology, Xiyuan Hospital, China Academy of Chinese Medical Sciences, Beijing, China; ^3^ Institute of Chinese Materia Medica, China Academy of Chinese Medical Sciences, Beijing, China

**Keywords:** Guanxinning, diabetes mellitus, myocardial ischemia reperfusion injury, oral microbiota, *Fusobacterium nucleatum*, PTEN

## Abstract

**Background:**

The incidence and severity of diabetic myocardial ischemia reperfusion injury (DMIRI) are increasing, highlighting the urgent need for effective prevention and treatment. Previous studies have revealed that specific oral microbiota (*Fusobacterium nucleatum*) are closely involved in DMIRI, potentially serving as therapeutic targets. Guanxinning (GXN) has shown significant efficacy in treating diabetic cardiomyopathy. However, its mechanisms of action regarding DMIRI and its relationship with specific microbiota remain to be elucidated.

**Proposal:**

This study investigates whether GXN alleviates DMIRI by modulating *F. nucleatum* and host interactions.

**Methods:**

The effects of GXN on cardiac injury, cardiac protein expression and the abundance of *F. nucleatum* were evaluated in C57BL/6 mice under both conventional and germ-free conditions. GWAS analysis was employed to identify potential mechanisms linking *F. nucleatum* and DMIRI. *Fusobacterium nucleatum* IgG levels were measured, and LC-MS/MS metabolomics along with Metorigin trace-ability analysis were conducted to validate the proposed mechanisms.

**Results:**

GXN treatment significantly reduced myocardial injury in diabetic mice and decreased oral *F. nucleatum* abundance, although its effects on other gut microbiota taxa were variable. Importantly, the cardioprotective efficacy of GXN was markedly attenuated under pseudo-germ-free conditions, suggesting that its benefits are at least partly microbiota-dependent. PI3K signaling pathway was identified as a central mediator of the microbiota interaction in DMIRI. Correspondingly, cardiac tissues from diabetic mice exhibited reduced expression of PTEN, consistent with pathway activation. Notably, *F. nucleatum* exposure elevated plasma levels of specific anti–*F. nucleatum* IgG antibodies and appeared to influence the host PI3K pathway through modulation of phenylalanine metabolism.

**Conclusion:**

GXN may alleviate DMIRI by targeting *F. nucleatum* and PTEN related pathways, offering new insights into microbiota-based cardio protection.

## Highlights

• Guanxinning alleviates diabetic myocardial ischemia reperfusion injury (DMIRI) by regulating *Fusobacterium nucleatum* as a key target.
• Oral *F. nucleatum* exacerbates DMIRI via PI3K-Akt pathway dysregulation.

## 1 Introduction

Diabetes mellitus (DM) and its complications represent a growing global public‐health burden, with rising incidence and associated morbidity and mortality (Deng et al., 2024; Magliano et al., 2021). Patients with diabetes are at significantly higher risk of myocardial ischemia reperfusion injury (MIRI) than non-diabetic individuals (Zhong et al., 2024). The mechanisms by which diabetes exacerbates vascular injury are multifactorial and complex (Li et al., 2023). Our previous work demonstrated that dysbiosis of the oral microbiota may mediate diabetes-induced MIRI (Li et al., 2024). In particular, diabetes promotes the proliferation of oral *Fusobacterium nucleatum* (*F. nucleatum*), a keystone pathogen that contributes to microbial imbalance and worsens MIRI. Elevated oral *F. nucleatum* is a hallmark of this dysbiosis (Li et al., 2024), and *F. nucleatum* has been shown to modulate cardiac miR-21 expression, thereby aggravating MIRI. Other studies have confirmed that *F. nucleatum* regulates the miR-21/PTEN (Li et al., 2022) and PI3K signaling pathways, promoting inflammation and contributing to atherosclerotic cardiovascular disease progression (Shen et al., 2024; Zhou et al., 2023). Thus, elucidating how modulation of *F. nucleatum* and its molecular targets can mitigate diabetic MIRI (DMIRI) may yield novel evidence to inform clinical therapies.

Traditional Chinese Medicine (TCM) holds significant promise for treating diabetic cardiovascular complications (Wei et al., 2023; Liu et al., 2021). Guanxinning (GXN) is an approved TCM formulation whose principal botanical drug include Danshen (*Salvia miltiorrhiza* Bunge) and Chuanxiong (*Ligusticum chuanxiong* Hort) (Pang et al., 2024). Multiple randomized controlled trials have demonstrated that GXN effectively attenuates residual inflammation in patients with stable coronary artery disease (Sun et al., 2019; Chen et al., 2024) and significantly relieves anginal symptoms in diabetic patients (Li Xiaoying et al., 2023). Early administration of GXN may therefore reduce the risk or severity of MIRI in diabetes. Preclinical pharmacological studies have shown that GXN exerts protective effects against MIRI (Wang et al., 2022; Xiao et al., 2022), attenuating inflammation and oxidative stress while promoting angiogenesis (Chen et al., 2022; Ling et al., 2021). In a porcine model of atherosclerosis, GXN protected against endothelial injury, reduced vascular inflammation, and decreased oxidative damage—effects hypothesized to involve modulation of gut microbiota composition and its metabolites (Yang et al., 2022).

Notably, the cardioprotective mechanisms of GXN differ between *in vitro* and *in vivo* models (Ling et al., 2021; Chen et al., 2023; Kumar et al., 2023), suggesting potential interactions with the oral microbiome and other environmental factors. Both Danshen and Chuanxiong have been reported to exhibit significant inhibitory effects against oral pathogens (Chen et al., 2021; Liu et al., 2025). We therefore hypothesize that GXN mitigates DMIRI through interactions with the oral microbiota, leading to modulation of systemic and myocardial inflammatory responses. However, the precise molecular targets and underlying mechanisms remain to be defined.

In the present study, we employ animal models to evaluate the effects of GXN on DMIRI under conventional and pseudo-germ-free conditions, assessing its capacity to suppress oral *F. nucleatum* and confer cardioprotection. Combined with bacterial genome-wide association studies (GWAS), *F. nucleatum* gavage models, *F. nucleatum*-IgG detection, and metabolomic tracing analysis (Yu et al., 2022), we confirm that *F. nucleatum* primarily influences host PI3K pathway-related metabolites rather than directly altering microbial metabolism. We further validate that the PI3K pathway–associated phosphatase PTEN is involved in DMIRI and demonstrate that GXN exhibits a regulatory effect on PTEN expression. To our knowledge, this is the first systematic investigation of GXN’s molecular mechanism in attenuating DMIRI via modulation of oral *F. nucleatum*, establishing a novel oral microbiome–cardiac axis as a TCM therapeutic strategy and identifying *F. nucleatum* and PTEN as potential biomarkers and pharmacological targets for clinical intervention.

## 2 Materials and methods

### 2.1 Plant materials and extract preparation

GXN extract powder was supplied by Zhengda Qingchunbao Co., Ltd. (NMPA registration no. Z20086359; batch no. 202202). The formulation comprises two medicinal botanical drug in a 1: 1 dry-weight ratio: *Salvia miltiorrhiza* Bunge [Lamiaceae; *Salviae miltiorrhizae* radix et rhizoma; common name: Danshen]; *Ligusticum chuanxiong* Hort. [Apiaceae; *Conioselinum anthriscoides* “Chuanxiong” rhizoma (syn. *Ligusticum chuanxiong* Hort.); common name: Chuanxiong].All botanical names and family assignments were validated against Kew’s Medicinal Plant Names Services (MPNS).

The preparation was provided as a 10-mesh extract powder. Quality control markers, determined by HPLC per the manufacturer’s certificate of analysis, were as follows: salvianolic acid B (water-soluble phenolic acid marker) ≥ 62.5 mg/g and ferulic acid (phenolic ester marker) ≥ 3.8 mg/g. No additional extraction or fractionation was performed; the phytochemical profile of the commercial GXN powder is therefore considered stable.

### 2.2 Animals and experimental design

**FIGURE 1:**
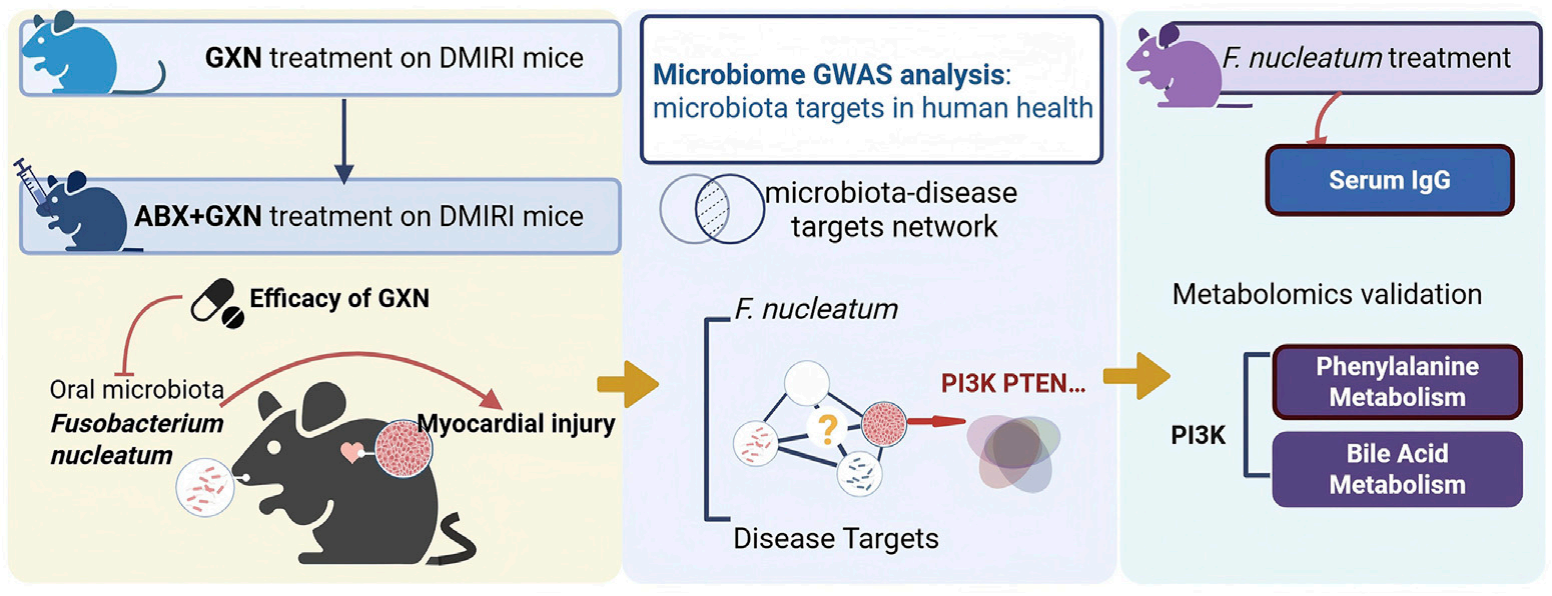
Workflow of the study. Abbreviations: GXN, Guanxinning extract powder; DMIRI, diabetic myocardial ischemia reperfusion injury; ABX: Antibiotics; GWAS: Genome-Wide Association Study; *F. uncleatum: Fusobacterium nucleatum.*

A total of 3 animal experiments were performed (Figure 1). Six-week-old male C57BL/6J mice were used for all animal experiments and housed in animal facilities with specific SPF levels. All mice were maintained under standard conditions (air humidity 40%–70%, ambient temperature 22°C ± 2°C, 12/12 h light/dark cycle). The mice were purchased from Spectrum (Beijing) Biotechnology Co., Ltd. (production license: SCXK (Beijing) 2019-0010). Additional reagents and mouse tissue examination protocols are detailed in Supplementary Material 1.

Experiment 1: C57BL/6 mice were divided into 6 groups to assess the impact of various interventions on MIRI. Initially, mice were categorized into a sham operation control group (CON) and a standard MIRI model group (MIRI). Additionally, a diabetes model was established by injecting streptozotocin (STZ) and further divided into 4 groups: diabetic MIRI group (DMIRI), diabetic group treated with dapagliflozin (Dapa), and groups receiving high-dose (GXN-H) and low-dose (GXN-L) GXN treatment, respectively. All mice underwent respective treatments for 6 weeks: CON, MIRI, DMIRI groups received an equivalent volume of water daily, Dapa group received 10 mg/kg of dapagliflozin per body weight daily; GXN-H and GXN-L received 1,200 mg/kg and 600 mg/kg of GXN extract powder solution per body weight daily, respectively (Pang et al., 2024). Throughout the experiment, the mice did not exhibit any severe toxic symptoms. After 6 weeks, except for the control group, all other groups underwent surgery to simulate MIRI by ligating the left anterior descending coronary artery for 30 min followed by 24 h of reperfusion.

Experiment 2: C57BL/6 mice were divided into 4 groups to evaluate the impact of different interventions on MIRI under pseudo-germ-free conditions. A diabetes model was established by injecting STZ and further divided into 4 groups, with all mice undergoing 6 weeks of intervention: antibiotic gavage plus the same medication regimen as in Experiment 1. After 6 weeks, all groups underwent surgery to simulate MIRI by ligating the left anterior descending coronary artery for 30 min followed by 24 h of reperfusion.

Experiment 3: C57BL/6 mice were divided into 3 groups to assess the impact of *F. nucleatum* intervention on MIRI. The groups were a blank control group (CON), a *F. nucleatum* gavage group (F.n), and a *F. nucleatum* gavage with MIRI group (F.n-MIRI). Gavage was administered for 6 weeks, with every other day administration for the first 2 weeks followed by once a week for the next 4 weeks to maintain microbial colonization (Liu et al., 2020). After 6 weeks, all groups underwent surgery to simulate MIRI by ligating the left anterior descending coronary artery for 30 min followed by 24 h of reperfusion.

### 2.3 Sample collection and processing

Mice were fasted for 4 h prior to sample collection. Blood glucose levels were determined from tail-tip blood samples to measure fasting glucose. Five mice from each group were randomly selected for TTC-Evans blue staining, while the remaining mice were used for other tissue sampling.

Blood Collection: Blood samples were collected via retro-orbital bleeding and processed to prepare plasma. Oral Microbiota Sampling (Dong et al., 2021): Mice were immobilized, and a sterile swab was gently inserted into the oral cavity, including the tongue (dorsal and ventral surfaces), gingiva, and saliva, to thoroughly collect oral microbiota. The swab tip was then placed into a sterile cryovial, rapidly frozen in liquid nitrogen, and stored at −80°C. Collection of Colonic Contents (Tang et al., 2019): After opening the abdominal cavity, the cecum was located, and colonic contents from the distal ileum and beyond were collected. Using sterile forceps, the distal ileum was gently squeezed to extrude the contents into a sterile cryovial, which was then rapidly frozen in liquid nitrogen and stored at −80°C. Heart Tissue Sampling: Heart tissues were excised and divided into the base and apex regions, using the coronary ligation site as a reference. Apex tissues were placed in cryovials, immediately frozen in liquid nitrogen, and stored at −80°C. The base tissues were fixed in 4% paraformaldehyde solution. Details are summarized in the Supplementary Material.

### 2.4 Pseudo-germ-free mice

In Experiment 2, to establish a pseudo-germ-free mouse model (Wang et al., 2019), experimental mice were subjected to a 6-week treatment with a cocktail of antibiotics via gavage (ampicillin 1 g/L, neomycin sulfate 1 g/L, vancomycin 0.5 g/L, metronidazole 1 g/L) while receiving drug intervention at a dose of 200 µL/day.

### 2.5 Western blot analysis

Total protein was extracted from frozen cardiac tissue homogenized in 1 mL lysis buffer on ice, incubated for 20 min with vortexing every 5 min, and centrifuged at 12,000 rpm for 10 min at 4°C. The supernatant was collected, recentrifuged, and stored at −80°C. Protein concentration was determined using the BCA assay. Equal amounts of protein (40 μg) were mixed with 5× loading buffer (1:4), boiled for 5 min, and loaded onto 15% SDS-PAGE gels. Electrophoresis was performed at 80 V (stacking gel) and 100 V (separating gel). Proteins were transferred to PVDF membranes pre-soaked in transfer buffer using a semi-dry transfer system at 30 mA for 90 min. Membranes were blocked with 5% non-fat milk in TBS-T for 1 h. After washing with TBS-T (10 min × 3), membranes were incubated with primary antibodies (PTEN 1:1,000; GAPDH 1:4,000) at 4°C overnight. Following additional washes, HRP-conjugated secondary antibodies (PTEN 1:2,000; GAPDH 1:5,000) were applied at 37°C for 1 h. Signals were detected using ECL and exposed to X-ray film. Band intensities were quantified using Image-Pro Plus (IPP) software.

### 2.6 GWAS analysis

We incorporated the SNP (single nucleotide polymorphism) information (chromosome number, position, beta value, p-value, etc.) concerning the trait *F. nucleatum* by retrieving the GWAS summary website (https://gwas.mrcieu.ac.uk/) through the “extract_instruments” function of TwosampleMR package in RStudio (Yang et al., 2023). When conducting the “extract_instruments” function, p1 and p2 were set as 0.005, r2 were set as 0.8, and others were complied with the default settings. Subsequently, filtered by criteria that pval.exposure < 1e-06, the pivotal SNPs were incorporated and queried in the NCBI Genome Data Viewer (https://www.ncbi.nlm.nih.gov/gdv/) to screen for their potential upstream and downstream proximity genes associated with *F. nucleatum*. These targets were further included to perform protein-protein network construction and enrichment analyses. Disease-related targets for T2DM and myocardial I/R injury were identified from GeneCards (Safran et al., 2010) and OMIM (Sayers et al., 2022). F.nucleatum SNPs-disease intersecting targets were mapped. PPI analysis was performed with STRING, and GO/KEGG enrichment analysis (Kanehisa and Goto, 2000; Kanehisa et al., 2021) was conducted using DAVID.

### 2.7 Bacterial strain cultivation

*F. nucleatum* was cultured in thioglycolate liquid medium. Prepare a test tube containing approximately 10 mL of liquid medium that has been preconditioned in an anaerobic environment for 24 h. Disinfect the surface of the bacterial ampoule, open it in a safety cabinet, and sterilize the top with an alcohol lamp. Quickly add sterile water to break the ampoule, then use forceps to fully open it. Transfer approximately 0.5 mL of the liquid medium into the freeze-dried tube to dissolve the bacteria thoroughly. Subsequently, transfer the mixture back into the test tube containing the liquid medium and mix well. Place the test tube under specified anaerobic conditions for cultivation. When the suspension reaches a turbidity corresponding to approximately 10^9 colony-forming units (CFU) per milliliter, the bacterial culture is considered ready for gavage administration.

### 2.8 Metabolomics analysis

Metabolites Extraction and Derivatization: Metabolites were first extracted and derivatized. Data acquisition was performed using an Ultra Performance Liquid Chromatography (UPLC) system (ExionLC™ AD, https://sciex.com.cn/) coupled with Tandem Mass Spectrometry (MS/MS) (QTRAP^®^ 6500+, https://sciex.com.cn/).

Mass Spectrometry Conditions: The electrospray ionization (ESI) source operated at a temperature of 550°C. The mass spectrometer voltage was set to 5,500 V in positive ion mode and −4,500 V in negative ion mode, with a curtain gas (CUR) pressure of 35 psi. Each ion pair was detected in the QTRAP 6500+ system based on optimized declustering potential (DP) and collision energy (CE).

Qualitative and Quantitative Analysis Principles: Qualitative analysis was performed by comparing the mass spectrometry data to a database constructed with known standards (Metware Database, MWDB). Quantitative analysis was conducted using the Multiple Reaction Monitoring (MRM) mode of triple quadrupole mass spectrometry.

In MRM mode, the quadrupole filters the precursor ion of the target metabolites, excluding ions of other molecular weights to minimize interference. The precursor ion is fragmented in the collision chamber to produce multiple fragment ions. Specific fragment ions are then selected by the triple quadrupole system, eliminating non-target ion interference to ensure precise and reproducible quantification.

### 2.9 MetOrigin trace-ability analysis

Analysis was conducted using plasma metabolomics data from mice. The microbial origin of metabolites was analyzed using MetOrigin (Yu et al., 2022) (https://metorigin.met-bioinformatics.cn/). Metabolites profiles and microbial abundance data were integrated to predict microbial contributions based on metabolic pathways. Analysis followed the standard workflow of the platform.

### 2.10 Statistical analysis

A Student’s t-test, two-way ANOVA followed by Sidak’s multiple comparison test, and a Mann–Whitney U test were conducted using GraphPad Prism (V9.5) and IBM SPSS Statistics (V26.0). Pearson’s chi-squared test was applied to analyze the differences in sex, drinking status, and follow-up rates across groups. The Adonis test was performed using R software. The Kruskal–Wallis test was used to assess the microbiota abundance. Spearman’s correlations and K-means clustering of metabolites were analyzed and visualized using R package.

## 3 Results

### 3.1 GXN regulates the oral *Fusobacterium nucleatum* and alleviates DMIRI

**FIGURE 2:**
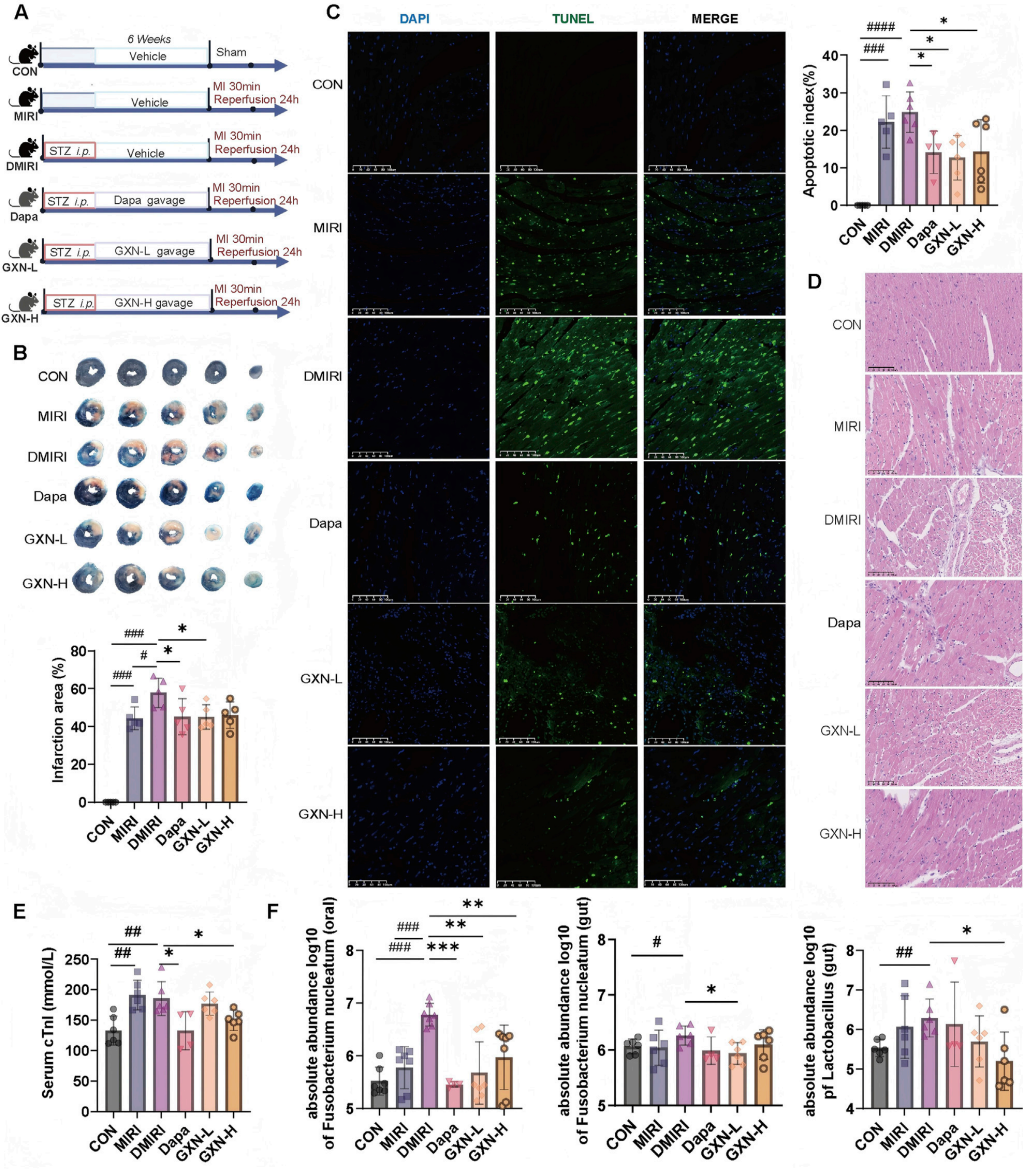
GXN regulates oral microbiota and alleviates DMIRI. **(A)** Schematic illustration of the experimental design. STZ: streptozotocin; MI: myocardial infarction (left anterior descending artery ligation); GXN: Guanxinning extract. **(B)** TTC staining showing the infarct area as a percentage of the left ventricular area in mice (N = 4–5). **(C)** Representative TUNEL staining of the left ventricle, with the bar plot showing the ratio of apoptotic cells (green) to total cells (blue and green) (magnification: ×20) (N = 5–6). **(D)** Representative H&E staining of the left ventricle (magnification: ×20) (N = 5). **(E)** Serum cTnI levels (N = 5–6). **(F)** Absolute abundance of *Fusobacterium nucleatum* (in oral and gut) and *Lactobacillus* (in gut) (N = 5–6). Abbreviations: CON, sham operated group; MIRI, MIRI group; DMIRI, DMIRI group; Dapa, dapagliflozin treatment group; GXN-L, low-dose GXN treatment group; GXN-H, high-dose GXN treatment group. #Compared with the CON group, *#P <* 0.05, *##P <* 0.01, *###P <* 0.001; * compared with the DMIRI group, *P < 0.05, **P < 0.01, ***P < 0.001.

Based on the disease mechanism of DMIRI involving oral microbiota, *F. nucleatum* has been identified as a pathogenic feature of DMIRI (Li et al., 2024). This study further investigated whether GXN selectively modulates *F. nucleatum* and whether it exerts cardioprotective effects against DMIRI. In a high-glucose model, GXN was administered via oral gavage to evaluate its prophylactic effect on MIRI (Figure 2A). Compared with the MIRI group, the DMIRI group exhibited more severe cardiac injury, as evidenced by TTC staining, hematoxylin-eosin (HE) staining, TUNEL staining, and elevated myocardial injury biomarkers (Figures 2B–E).

Compared with the DMIRI group, the dapagliflozin-treated group (Dapa), the low-dose GXN group (GXN-L) showed significantly smaller infarction areas (*P <* 0.05; Figure 2B); Dapa, GXN-L and the high-dose GXN group (GXN-H) reduced cardiomyocyte apoptosis (*P <* 0.05; Figure 2C). HE staining revealed improved cardiomyocyte alignment, decreased inflammatory infiltration, and more compact myocardial architecture in the treatment groups (Figure 2D). Furthermore, serum cardiac troponin I (cTnI) levels were significantly lower in the Dapa and GXN-H groups compared with the DMIRI group (*P <* 0.05; Figure 2E). These findings indicate that both low and high doses of GXN confer protective effects against DMIRI.

Oral *F. nucleatum* abundance was significantly increased in the MIRI group compared with the control (CON) group (*P <* 0.001; Figure 2F), whereas no significant differences were observed in gut *F. nucleatum* or *Lactobacillus* levels (*P >* 0.05). Compared with the MIRI group, the DMIRI group showed further elevation in oral *F. nucleatum* abundance (*P <* 0.001; Figure 2F), again without changes in gut *F. nucleatum* or *Lactobacillus*. Notably, oral *F. nucleatum* abundance was significantly reduced in the Dapa, GXN-L, and GXN-H groups compared with the DMIRI group (*P <* 0.001, *P <* 0.01, and *P <* 0.01, respectively; Figure 2F), suggesting a marked regulatory effect of GXN on oral *F. nucleatum*. Additionally, GXN-L reduced gut *F. nucleatum* abundance (*P <* 0.05), while GXN-H decreased gut *Lactobacillus* abundance (*P <* 0.05). Dapagliflozin showed no significant effect on either bacterial genus (Figure 2F). These results suggest that diabetes and MIRI promote the enrichment of oral *F. nucleatum*, and that GXN, in both low and high doses, effectively attenuates its abundance. GXN also modulates gut microbial composition, though the direction of change varies by dose and bacterial species.

### 3.2 GXN attenuates DMIRI via modulation of oral *Fusobacterium nucleatum*

**FIGURE 3:**
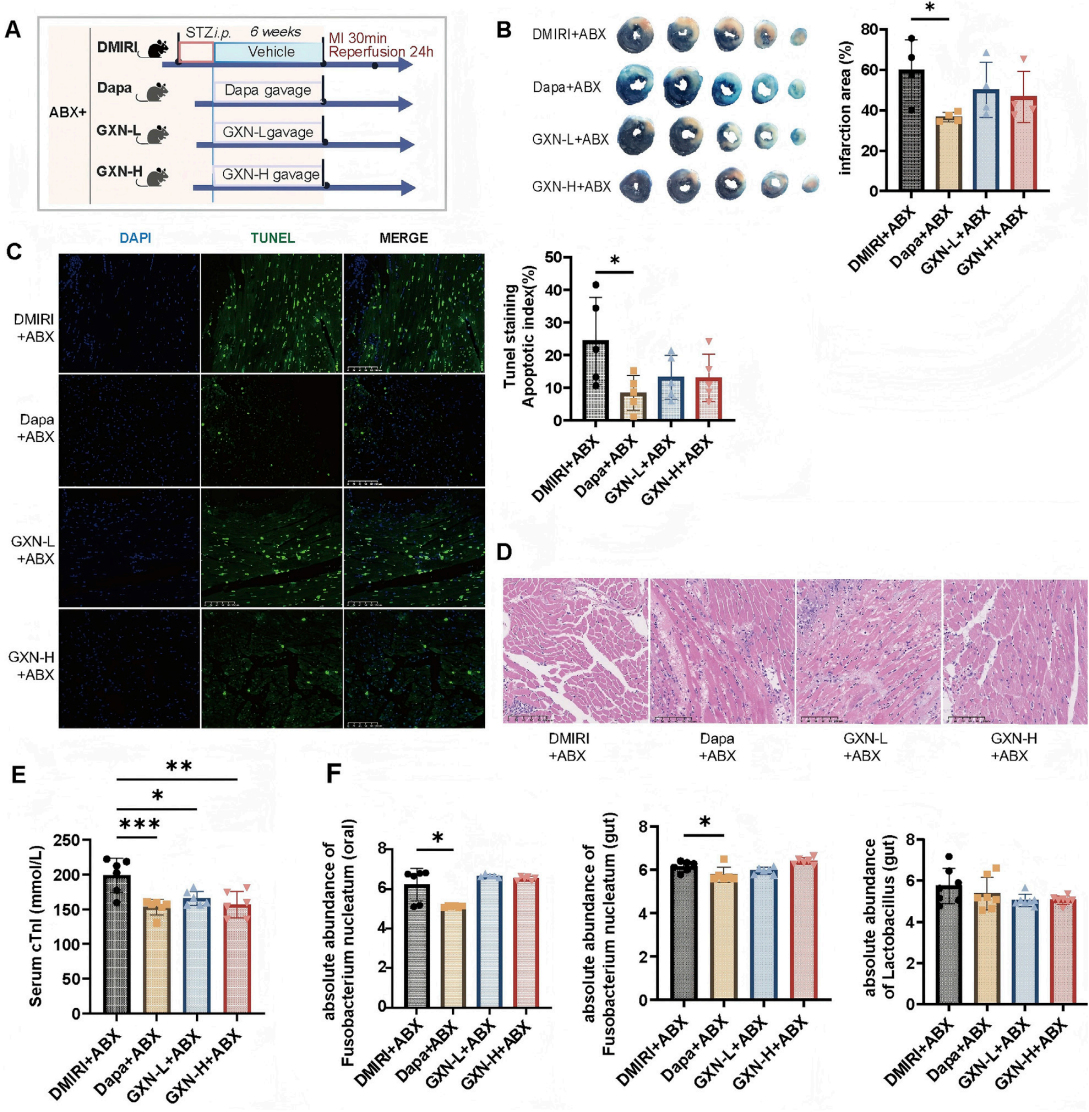
Pseudo-Germ-Free Conditions Diminish GXN’s Effectiveness Against DMIRI. **(A)** Schematic illustration of the experimental design. ABX: antibiotics; STZ: streptozotocin; MI: myocardial infarction (left anterior descending artery ligation); GXN: Guanxinning extract. **(B)** TTC staining showing the infarct area as a percentage of the left ventricular area in mice (N = 5). **(C)** Representative TUNEL staining of the left ventricle, with the bar plot showing the ratio of apoptotic cells (green) to total cells (blue and green) (magnification: ×20) (N = 5). **(D)** Representative H&E staining of the left ventricle (magnification: ×20) (N = 5). **(E)** Serum cTnI levels (N = 6). **(F)** Absolute abundance of *Fusobacterium nucleatum* (in oral and gut) and *Lactobacillus* (in gut) (N = 6–8). Abbreviations: DMIRI + ABX: Antibiotic-induced pseudo-germ-free DMIRI injury group; Dapa + ABX: Antibiotic-induced pseudo-germ-free dapagliflozin treatment group; GXN-L + ABX: Antibiotic-induced pseudo-germ-free low-dose GXN treatment group; GXN-H + ABX: Antibiotic-induced pseudo-germ-free high-dose GXN treatment group. *compared with the DMIRI + ABX group, *P < 0.05, **P < 0.01, ***P < 0.001.

To further verify whether the efficacy of GXN depends on its impact on *F. nucleatum*, we administered broad-spectrum antibiotics during GXN gavage in mice to establish pseudo-sterile conditions and assess the extent of DMIRI (Figure 3A). Results showed that under pseudo-sterile conditions, the positive control drug dapagliflozin still exhibited significant myocardial protection, while the efficacy of GXN was significantly weakened (Figures 3B–E). TTC staining and TUNEL staining showed that both the GXN-L + ABX and GXN-H + ABX groups did not significantly reduce the myocardial infarction area and apoptosis levels in DMIRI mice (*P >* 0.05, Figures 3B,C). HE staining results showed that the myocardium in the DMIRI + ABX group exhibited unclear myofibrils with extensive inflammatory infiltration. In contrast, the myocardium in the Dapa + ABX, GXN-L + ABX, and GXN-H + ABX groups had clearer myofibrils and more orderly cell arrangement. However, the GXN-L + ABX and GXN-H + ABX groups did not significantly alleviate the inflammatory infiltration (Figure 3D). Compared with the DMIRI + ABX group, the serum cTnI levels in the Dapa + ABX group and the GXN-L + ABX and GXN-H + ABX groups were significantly reduced (*P <* 0.05; Figure 3E), indicating that the myocardial protective effect of GXN was weakened under pseudo-sterile conditions.

qPCR analysis showed no significant differences in the abundance of oral *F. nucleatum* between the GXN-L + ABX, GXN-H + ABX, and DMIRI + ABX groups (*P >* 0.05, Figure 3F), nor in the abundance of gut *F. nucleatum* or *Lactobacillus* (*P >* 0.05, Figure 3F). In contrast, the Dapa + ABX group showed a significant reduction in oral and gut *F. nucleatum* abundance (*P <* 0.05, Figure 3F). These findings suggest that the efficacy of GXN is associated with the microbial environment, particularly the presence of *F. nucleatum*, and may alleviate DMIRI by modulating its abundance. Therefore, further investigation is needed to elucidate the role of *F. nucleatum* in host DMIRI pathology and to identify potential therapeutic targets.

### 3.3 PTEN involvement in *Fusobacterium nucleatum*–Associated PI3K signaling in DMIRI

**FIGURE 4:**
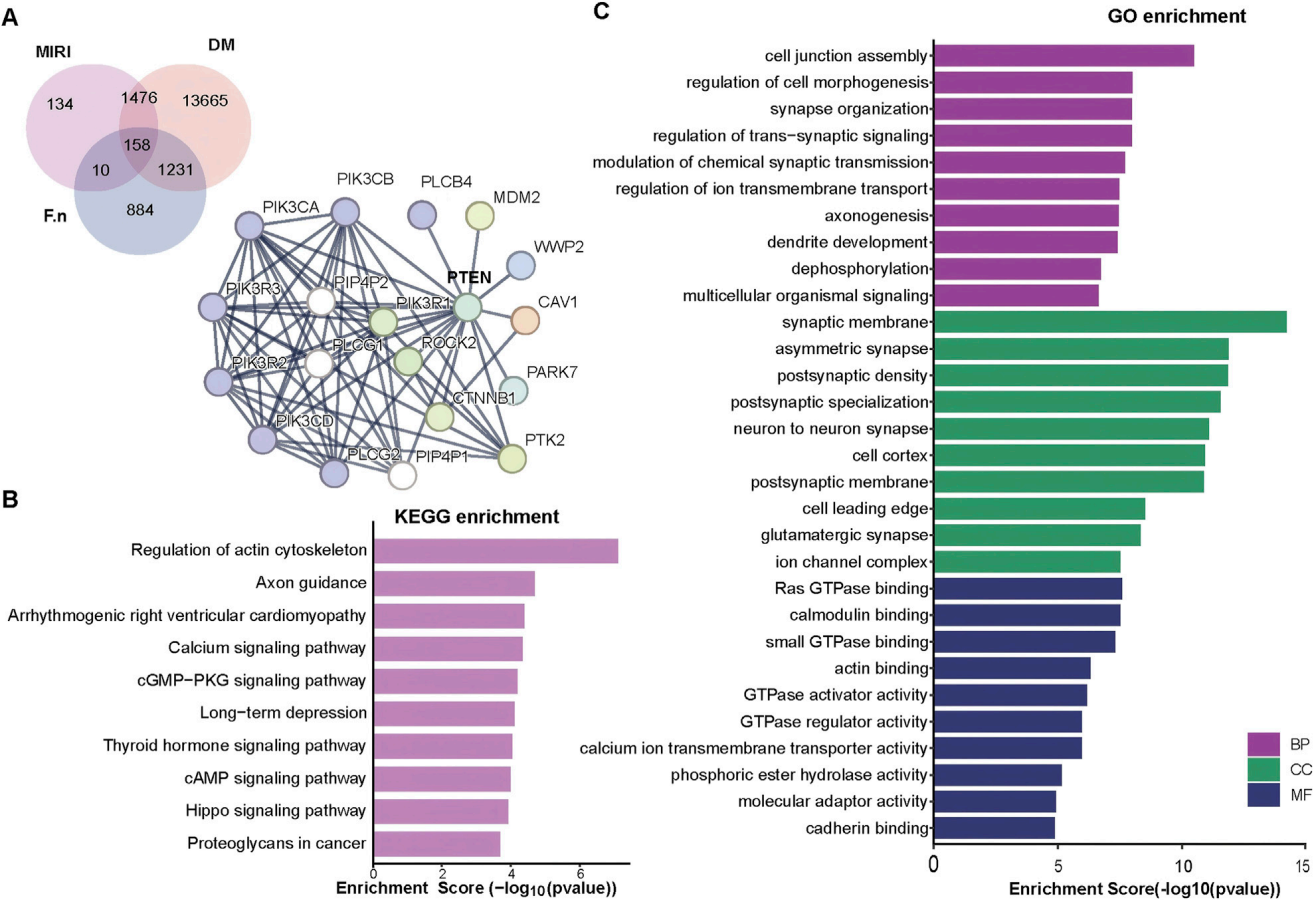
*Fusobacterium nucleatum* associated SNPs host gene functions protein interactions and their relevance to DMIRI. **(A)** Venn diagram depicting the overlap of *Fusobacterium nucleatum*-related SNP genes, DM and MIRI targets; Protein-protein interaction (PPI) network of the 158 overlapping genes. **(B,C)** GO and KEGG enrichment analyses of core PPI genes.

Given the complex relationship between the microbiota and disease, GWAS were first employed to identify genetic factors potentially involved in microbial regulation. A total of 16,530 and 1,778 candidate genes associated with DM and MIRI, respectively, were retrieved from the GeneCards and OMIM databases. SNP data associated with *F. nucleatum* were extracted from the GWAS Catalog, and variants with a genome-wide significance threshold of *P <* 1 × 10^−6^ were selected. Chromosomal loci corresponding to these SNPs were annotated using the Genome Data Viewer in the NCBI database to identify nearby genes as potential microbial target genes. This analysis yielded 2,283 genes potentially influenced by *F. nucleatum*. A Venn diagram comparing these genes with those associated with DM and MIRI identified 158 overlapping genes (Figure 4A). Protein-protein interaction (PPI) networks were constructed using the STRING database, revealing a core interaction module enriched in genes involved in the PI3K signaling pathway, including PTEN, PIK3R1, PIK3R2, PIK3R3, etc., (Figure 4A). GO and KEGG pathway enrichment analyses further demonstrated that these overlapping genes were predominantly associated with processes such as regulation of the actin cytoskeleton (Figures 4B,C).

**FIGURE 5:**
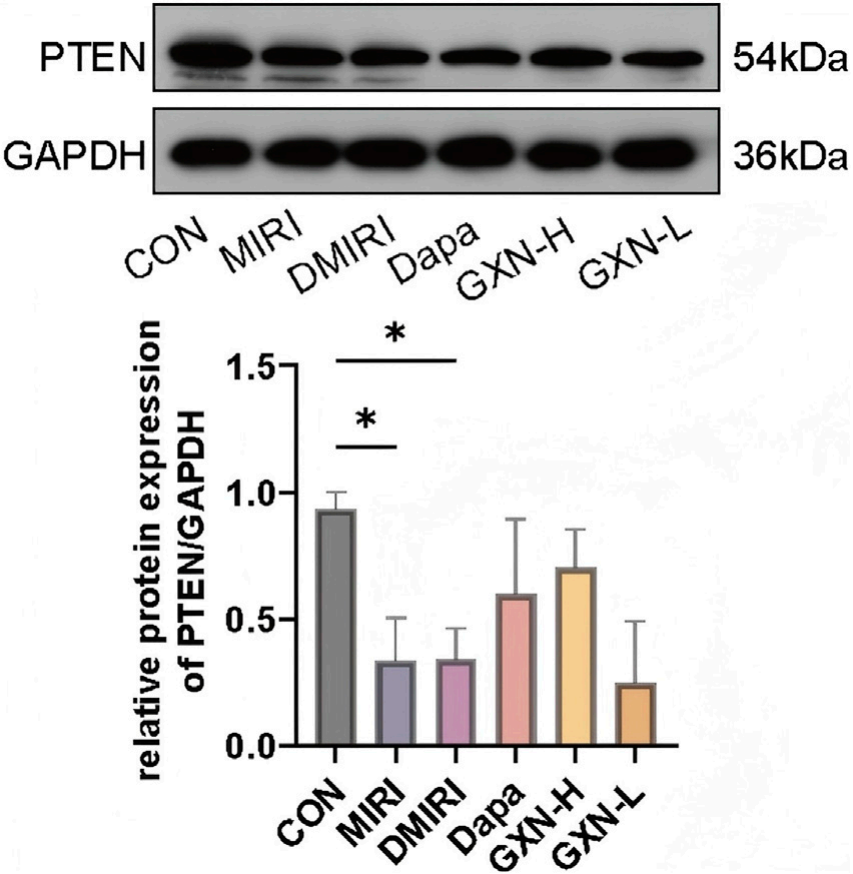
PTEN protein expression in cardiac tissue. *P < 0.05.

To validate these findings, we assessed PTEN protein expression in cardiac tissue. Compared to the CON group, both the MIRI and DMIRI groups showed significantly reduced PTEN expression in myocardial tissue (*P <* 0.05; Figure 5). Although an increasing trend in PTEN expression was observed in the GXN-H group compared to the DMIRI group, the difference was not statistically significant (*P >* 0.05; Figure 5). These results suggest that PTEN may be a potential target influenced by *F. nucleatum* in the context of DMIRI.

### 3.4 *Fusobacterium nucleatum* alters PI3K-Akt signaling and regulates metabolite profiles

**FIGURE 6:**
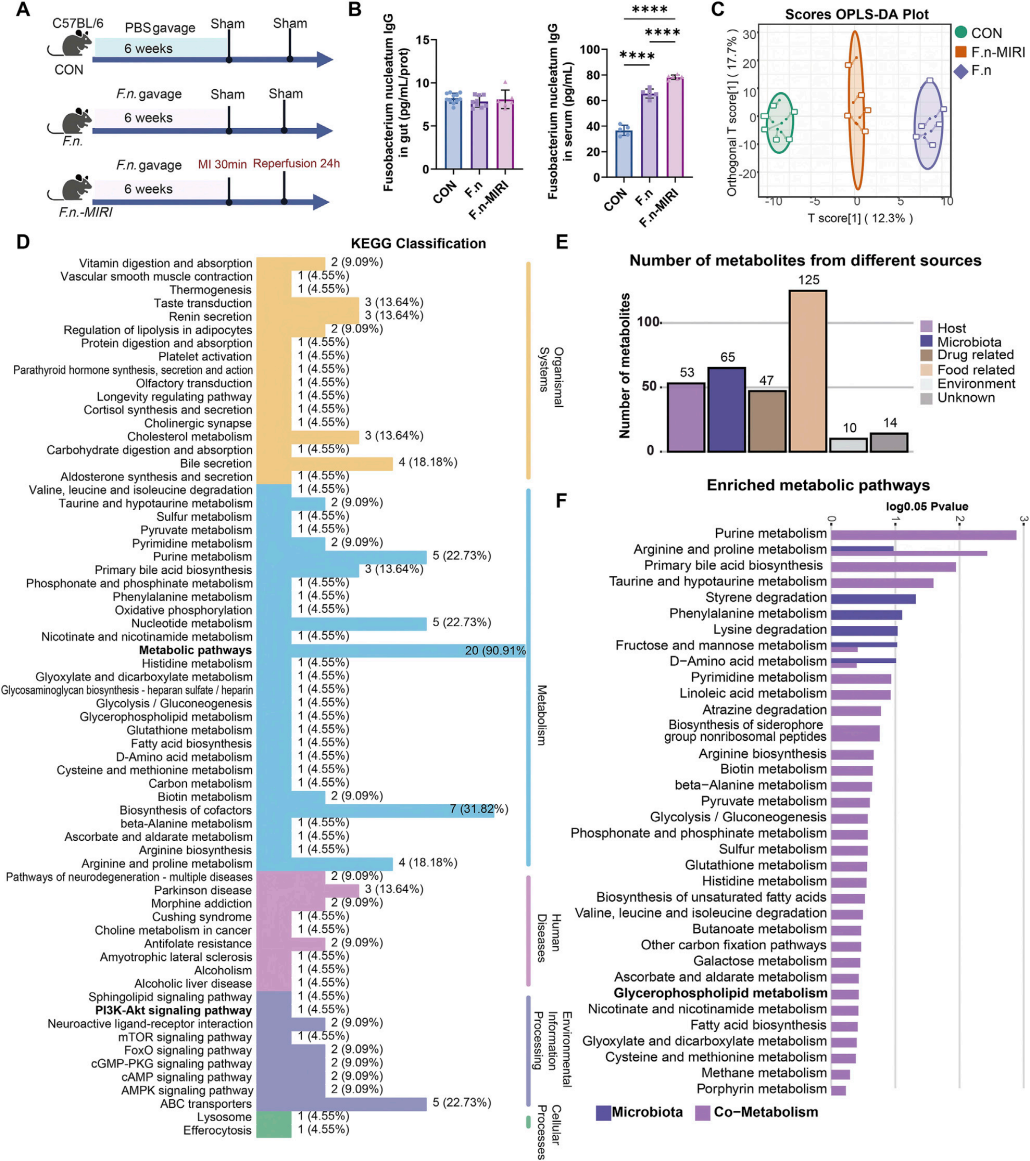
*Fusobacterium nucleatum*-Induced Plasma Metabolic Changes in MIRI Mice. **(A)** Schematic illustration of the experimental design. **(B)** *Fusobacterium nucleatum* IgG antibody levels in the gut (N = 6–11) and plasma (N = 5–6). **(C)** OPLS-DA score plot of all groups. **(D)** KEGG pathway annotation of metabolic pathways. **(E)** Bar plot showing microbiota-derived and host-derived metabolites. **(F)** Functional annotation of microbiota-derived and host-derived metabolism. Abbreviations: CON, vehicle gavage + sham surgery group; F.n: *Fusobacterium nucleatum* gavage + sham surgery group; F.n-MIRI, *Fusobacterium nucleatum* gavage + MIRI group.

We further validated the mechanisms by which *F. nucleatum* affects the human body (Figure 6A). We evaluated immune activation by measuring the levels of *F. nucleatum*-specific IgG antibodies in the colon and plasma. In the *F. nucleatum* gavage group (F.n) and the MIRI model group (F.n-MIRI), colon tissue levels of *F. nucleatum*-specific IgG antibodies did not show significant changes compared to the control (CON) group (*P >* 0.05), whereas plasma levels were significantly elevated (*P <* 0.05) (Figure 6B). These results suggest that *F. nucleatum* modulates systemic immune responses without affecting local mucosal immunity in the colon.

Therefore, we hypothesize that *F. nucleatum* does not compromise the gut barrier directly, but rather influences disease progression through systemic effects mediated by metabolites and other small molecules. The results of differential metabolite screening and identification using OPLS-DA analysis demonstrated clear separation among the CON, F.n, and F.n-MIRI groups, indicating that *F. nucleatum* significantly alters host plasma metabolism (Figure 6C). We identified specific differential metabolites across these groups, particularly amino acid derivatives and bile acid metabolites (Supplementary Figure S1). KEGG pathway enrichment analysis revealed that these metabolites were involved in various host metabolic pathways, including the PI3K-Akt inflammatory signaling pathway (Figure 6D), supporting GWAS-based findings that *F. nucleatum* may modulate host PI3K pathway activity.

Through the tracing of metabolites, we clearly distinguished the sources of the differential metabolites: 65 metabolites may originated from the microbiota, while 53 may originated from the host’s own metabolism (Figure 6E). This result reveals the potential mechanism by which *F. nucleatum* impacts the host’s pathological process by modulating metabolites. Functional annotations showed that purine metabolism and primary bile acid metabolism were the primary metabolic pathways sourced from the mixed origin, while phenylalanine metabolism was sourced from the microbiota (Figure 6F; Supplementary Figure S2). Therefore, *F. nucleatum* may influence the host’s own bile acid metabolism or promote DMIRI by affecting the metabolism of phenylalanine in the microbiota. In summary, GXN may alleviate DMIRI by inhibiting the *F. nucleatum*-Cardiac PTEN. This process may be related to the regulation of the bile acids and phenylalanine metabolism.

## 4 Discussion

Diabetes significantly increases the risk of MIRI (Wereski et al., 2022). Our study reveals a novel microbiota-mediated mechanism underlying DMIRI (Zhang Y. et al., 2024). Diabetes-induced oral microbiome dysbiosis, characterized by an overgrowth of *Fusobacterium nucleatum*, aggravates MIRI. In hyperglycemic mice, augmented oral *F. nucleatum* was associated with increased infarct size and inflammation (Li et al., 2024). Through this novel mechanism of DMIRI, regulating oral *F. nucleatum* may become a key strategy.

In traditional Chinese medicine, diabetic heart disease is often attributed to “blood stasis” syndrome, and blood-activating formulas like GXN are classically used to improve circulation (Author Anonymous, 2022; He et al., 2023). GXN has shown remarkable clinical efficacy and possesses multi-target and multi-pathway cardiovascular protective effects (Wang et al., 2022; Wang W. et al., 2024; Wang Y. et al., 2024). Our findings extend this perspective by showing that GXN’s cardioprotective effect involves regulation of the oral microbiota. Specifically, GXN treatment markedly reduced the oral abundance of *F. nucleatum*, as confirmed by *F. nucleatum*–specific qPCR. In conventional (microbiota-intact) diabetic mice, GXN alleviated DMIRI and restored cardiac function, whereas in antibiotic-depleted (pseudo-sterile) mice the protective effect of GXN was largely abolished.

Therefore, we continued to explore in depth the specific mechanism of *F. nucleatum* on the host. Through GWAS analysis, we identified 158 genes commonly associated with *F. nucleatum*, DM, and MIRI. Among these, PTEN and several PI3K pathway–related genes (e.g., *PIK3R1/2/3*) formed the core interaction network. As a negative regulator of the PI3K-Akt signaling pathway (Zhang J. et al., 2024), PTEN is downregulated in diabetic hearts, which may lead to Akt hyperactivation, exacerbating oxidative stress and apoptosis. *Fusobacterium nucleatum* may interfere with host PTEN expression by secreting metabolic products (such as phenylalanine derivatives) or outer membrane proteins, thereby amplifying the proinflammatory signals of the PI3K-Akt pathway. Western blot analysis confirmed a reduction of cardiac PTEN protein levels in DMIRI, and high-dose GXN has the potential to modulate PTEN expression.

We further explored whether *F. nucleatum* directly impacts host metabolism and immunity. Results revealed that it promotes systemic immune responses and alters host plasma amino acid and bile acid metabolic profiles (Liu et al., 2020). Notably, phenylalanine-derived metabolites such as phenylacetylglutamine were significantly elevated in the *F. nucleatum* intervention group, which may promote neutrophil infiltration and the release of proinflammatory cytokines by activating the PI3K-Akt pathway (Hooppaw et al., 2022; Zhou et al., 2022). On the other hand, host-derived metabolic disturbances are closely linked to myocardial energy metabolism dysfunction (Chang et al., 2024; Chang et al., 2023). Metabolic tracing analysis indicated that *F. nucleatum* primarily affects host metabolic activity, providing a theoretical basis for therapeutic strategies targeting host metabolic regulators (e.g., PTEN) rather than direct antimicrobial interventions.

Nevertheless, this study has several limitations. Although we observed altered PTEN expression in diabetic hearts, its functional role was not validated through gene knockout or overexpression experiments. In addition, antibiotic pretreatment may have nonspecifically disrupted other commensal microbiota, potentially compromising the reliability of the results. Finally, the exact role of differential metabolites such as phenylacetylglutamine in modulating the PI3K signaling pathway requires further validation using *in vitro* cell models (Kumar et al., 2023; Huang et al., 2024). Clinical validation of GXN in diverse populations with metabolic cardiovascular diseases, including assessments of its efficacy and safety, should also be prioritized in future studies. Rapidly advancing technologies such as microbiota single-cell genomics (Jia et al., 2024; Lloréns-Rico et al., 2022), and organoids (Arnauts et al., 2022; Puschhof et al., 2021) are offer promising opportunities to unravel the complex interactions among drugs, microbiota, and the host. These approaches could be utilized to further elucidate the molecular mechanisms by which GXN and other natural medicines regulate the microbiota, providing innovative therapeutic strategies for DMIRI and other microbiota-associated diseases. Importantly, this study lays foundational evidence for the microbiota–host interaction as a novel therapeutic axis in metabolic cardiovascular disease.

## Data Availability

The original contributions presented in the study are included in the article/Supplementary Material, further inquiries can be directed to the corresponding authors.

## Supplementary material

SUPPLEMENTARY FIGURE S1 Plasma metabolomic correlations and clustering in MIRI mice under the influence of *Fusobacterium nucleatum*. **(A)** Sankey diagrams showing significant metabolite crosstalk between CON vs. F.n (left), significant metabolite crosstalk between F.n vs. F.n-MIRI (right).**(B)** Heatmap displaying trends of differential metabolites among CON, F.n, and F.n-MIRI. **(C)** K-means clustering of metabolites, illustrating group-specific variation trends.

SUPPLEMENTARY FIGURE S2
*Fusobacterium nucleatum* participates in the phenylalanine metabolism pathway (ko00643_R02540.html).
