# Supplementary figures and images for: Guanxinning attenuates diabetic myocardial ischemia–reperfusion injury by targeting oral Fusobacterium nucleatum and modulating PTEN signaling
Source: Front Pharmacol. 2025 Jun 19;16:1581413. doi: 10.3389/fphar.2025.1581413 (PMC12223323; doi:10.3389/fphar.2025.1581413)

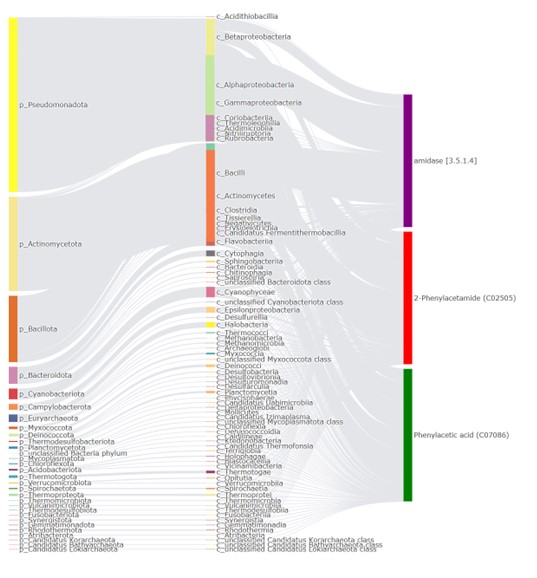

Supplement: Supplementary file 2 [file Image2.JPEG]

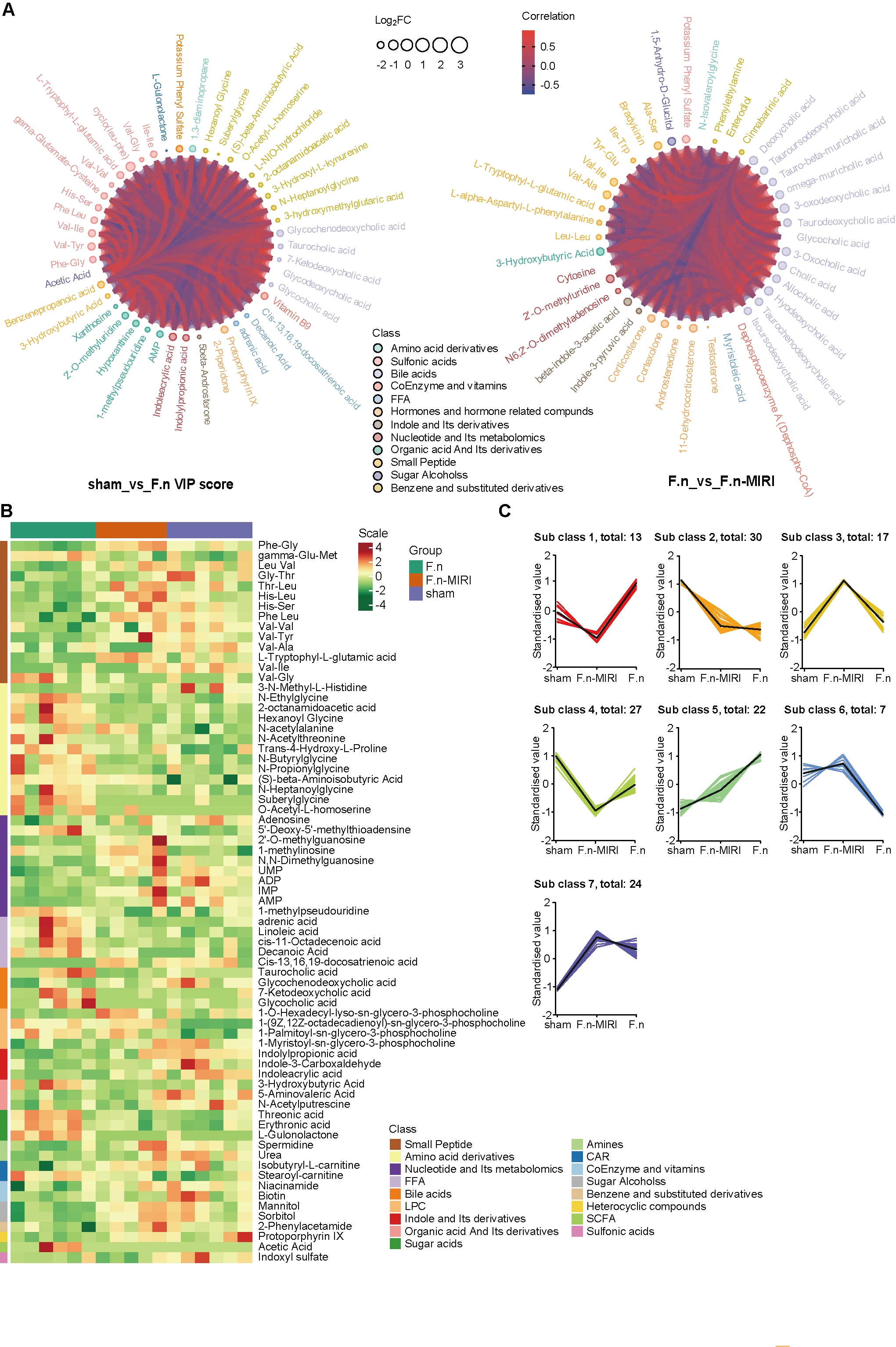

Supplement: Supplementary file 4 [file Image1.PNG]
