# Supplementary material for: Guanxinning attenuates diabetic myocardial ischemia–reperfusion injury by targeting oral Fusobacterium nucleatum and modulating PTEN signaling
Source: Front Pharmacol. 2025 Jun 19;16:1581413. doi: 10.3389/fphar.2025.1581413 (PMC12223323; doi:10.3389/fphar.2025.1581413)
Supplement: Supplementary file 5 [file DataSheet1.DOCX]

**Supplementary file1 Detailed methods**

**Materials and methods**

**Animal modeling**

**Diabetes Modeling**

C57BL/6J mice, maintained on a standard diet, were induced with diabetes by intraperitoneal injection of 50 mg/kg streptozotocin (STZ) for five consecutive days. Fasting blood glucose levels were monitored continuously for one week after induction. A fasting blood glucose level exceeding 200 mg/dL within the first week confirmed the successful establishment of the diabetes model.

**Pseudo-sterile Modeling**

Broad-spectrum Antibiotic (ABX) Regimen: A solution containing ampicillin (1 g/L) (Shanghai Aladdin Biochemical Technology Co.,Ltd., China (A105483)), neomycin sulfate (1 g/L) (Sigma-Aldrich, USA (N6386)), metronidazole (1 g/L) (Sigma-Aldrich, USA (M1547)), and vancomycin (0.5 g/L) (Shanghai Aladdin Biochemical Technology Co.,Ltd., China (V105495)) was prepared. The solution was administered once daily via gavage for a duration of six weeks, prepared freshly each day[11].

**Myocardial Ischemia-Reperfusion Injury (MIRI) Modeling**

Preoperative: Weigh and record the weight of all animals. Prepare animals for surgery by shaving the fur, induce anesthesia using isoflurane, and position the animals on a small animal surgical table in a supine position with limbs secured using 3M tape after the animals exhibit anesthesia responses such as disappearing corneal reflex and decreased muscle strength. After ensuring deep anesthesia, install the electrocardiogram monitor. Intraoperative: Disinfect the local skin, make an incision between the ribs after draping, bluntly dissect the muscles, open the pericardium, and expose the heart. Extend the heart outside the body, exposing the left anterior descending coronary artery. Ligature is applied approximately 1-2 mm from the bottom edge of the left atrial appendage using a 6-0 sterile silk thread. After confirming a whitening of the heart base, promptly close the chest, squeeze to prevent pneumothorax, and suture the muscles and skin layer by layer, inducing ischemia in the anterior wall of the left ventricle. After 30 minutes of ischemia, a second thoracotomy is performed, the ligature is released to restore blood flow to the left anterior descending coronary artery, and the chest is promptly closed and sutured to prevent pneumothorax.

**Detection Methods**

**TTC-Evans Blue Staining**

Heart slices were immersed in 1% 2,3,5-Triphenyl-2H-tetrazolium chloride (TTC) (Sigma-Aldrich, USA (T8877-50G)) in the dark. Blue areas represent the non-ischemic zone, red areas indicate the myocardial ischemic at-risk zone, and white areas indicate the myocardial infarction zone. The fixed tissue was photographed using a digital camera, and Image-ProPlus 6.0 software (National Institutes of Health, Bethesda, USA) was used for assessment, calculating the myocardial infarction area (%).

**Hematoxylin-Eosin（HE）Staining**

Collection of cardiac samples with a thickness of 3 mm, fixed with 4% paraformaldehyde and embedded in paraffin. Cross sections were stained with hematoxylin and eosin (Boruijie Technology Co., Ltd., China (DH0001/0405A17), Beijing Chemical Plant, China (20150915)). All images were captured using Leica DMi8 microscope.

**Tunel Staining**

The heart samples were separated and fixed in 10% phosphate-buffered formalin for 24 h, subsequently embedded in paraffin, sliced (4–5 μm). Terminal deoxynucleotidyl transferase-mediated dexoxyuridine triphosphate nick-end labeling (TUNEL) staining was performed using the TUNEL BrightGreen Apoptosis Detection Kit (Roche, Swiss) following manufacturer’s instructions. Apoptotic nuclei were labeled with green fluorescein staining and total cardiomyocyte nuclei were marked with DAPI. The pictures of heart tissues were viewed by confocal microscopy. Rate of apoptosis was displayed as ratio of TUNEL positive nuclei to DAPI-stained nuclei.

**Enzyme-linked immunosorbent assay (ELISA) analysis**

After treatment, blood samples were collected. Serum levels of cardiac troponin I (cTnI) (mm-0791M1) and insulin (mm-0579M1) were measured by the corresponding ELISA Kits (Jiangsu Meimian Industrial Co., Ltd, China), according to manufacturer’s instruction.

**qPCR sequencing primer sequence in validation cohort**

**Table1. Primer design**

| **Full name of primers** | **Primer sequence（5' to 3'）** | |
| --- | --- | --- |
| *Fusobacterium nucleatum* | upstream primer | CAACCATTACTTTAACTCTACCATGTTCA |
| *Fusobacterium nucleatum* | downstream primer | GTTGACTTTACAGAAGGAGATTATGTAAAAATC |
| *Latcobacillus* | upstream primer | GAGGCAGCAGTAGGGAATCTTC |
| *Latcobacillus* | downstream primer | GGCCAGTTACTACCTCTATCCTTCTTC |

**Pharmacological Network Data Sources and Research Methods**

**Collection of GXN Chemical Components and Target Prediction**

Using the Traditional Chinese Medicine Database and Analysis Platform (TCMSP, https://tcmsp-e.com/), the active components of Salvia miltiorrhiza (Danshen) and Ligusticum chuanxiong (Chuanxiong) were retrieved under the conditions of oral bioavailability (OB) ≥ 30% and drug-likeness (DL) ≥ 0.18. The active components were screened, and duplicate or invalid targets were removed. The collected targets were standardized using the UniProt database (https://www.uniprot.org/), non-human genes were excluded, and redundant entries were deleted to obtain standardized gene names.

**Acquisition of Targets Related to Diabetes Mellitus and Myocardial Ischemia-Reperfusion Injury**

Keywords such as "type 2 diabetes mellitus" and "myocardial ischemic reperfusion injury" were searched in the GeneCards (https://www.genecards.org/) and OMIM (https://www.omim.org/) databases. The resulting disease-related targets were consolidated into an Excel file, duplicates were removed, and the targets were calibrated using the UniProt database to obtain standardized disease target gene information.

**Drug-Disease Target Prediction Results**

The drug component targets and disease targets were mapped against each other, and the overlapping genes were visualized using a Venn diagram. The "drug-component-target" network was constructed using Cytoscape 3.7.2 software.

**Construction of Protein-Protein Interaction (PPI) Network**

To further investigate the interactions between proteins targeted by GXN in the treatment of type 2 diabetes mellitus and myocardial ischemia-reperfusion injury, the intersecting genes were uploaded to the STRING database (https://string-db.org/) for PPI network construction. The species was set to "Homo sapiens," and the minimum interaction score was set to 0.4 to ensure reliability. Other parameters were kept as default. The results were saved in TSV format and imported into Cytoscape 3.7.2 for network analysis (Cytoscape → Tools → Network Analyzer → Network Analysis → Analyze Network). The network analysis results were saved, with node size and color representing the degree value (larger nodes indicate higher degrees) and edge thickness representing the combined score (thicker edges indicate higher scores). Core targets were selected to create the PPI network diagram.

**GO Enrichment Analysis and KEGG Pathway Analysis**

The intersecting genes of drug-disease targets were uploaded to the DAVID database (https://david.ncifcrf.gov/summary.jsp), selecting "OFFICIAL_GENE_SYMBOL" as the gene identifier and "Homo sapiens" as the species. Using DAVID 6.8, Gene Ontology (GO) analysis was performed to investigate the biological processes (BP), cellular components (CC), and molecular functions (MF) of the therapeutic targets of GXN in treating type 2 diabetes mellitus and myocardial ischemia-reperfusion injury. To elucidate the mechanisms of GXN, KEGG pathway enrichment analysis was conducted. The top 10 GO terms (BP, CC, MF) and 20 KEGG pathways related to diabetes and myocardial ischemia-reperfusion injury (P < 0.01) were selected as the primary gene functions and signaling pathways, predicting the mechanism of GXN in treating these conditions.
